# Foreign Body in the Masticatory Space as a Rare Complication of Orthognathic Surgery and Associated Dental Procedures: A Case Series and Literature Review

**DOI:** 10.3390/jcm14155234

**Published:** 2025-07-24

**Authors:** Andrea Frosolini, Antonino Ungaro, Flavia Cascino, Linda Latini, Valerio Ramieri, Paolo Gennaro, Guido Gabriele

**Affiliations:** 1 Maxillofacial Surgery Unit, Department of Medical Biotechnologies, University of Siena, 53100 Siena, Italy; andreafrosolini@gmail.com (A.F.); ungaro@student.unisi.it (A.U.); flaviacascino@hotmail.com (F.C.); gennaro2@unisi.it (P.G.); 2 Ortognatica Roma, Via Nomentana 311, 00137 Roma, Italy; valerioramieri@gmail.com

**Keywords:** foreign bodies, masticatory muscles, orthognathic surgery, postoperative complications, computed tomography, surgical instrumentation

## Abstract

**Background**: Foreign bodies (FBs) within the masticatory space are a rare but significant complication of oral and maxillofacial procedures. Despite advancements in orthognathic surgery, instrument breakage and accidental loss can lead to challenging secondary procedures. Clinical experience with retained foreign bodies in the masticatory space following orthognathic surgery and related dental procedures is summarized. **Methods**: A retrospective search was conducted in the surgical and radiological database of a tertiary referral center for maxillofacial surgery, covering procedures from January 2017 to December 2024. Patients were included if they had undergone orthognathic surgery and presented with a retained FB in the masticatory space confirmed through imaging. Clinical records, operative notes, imaging studies, and follow-up data were reviewed. **Results**: Out of 2092 procedures, four patients (0.19%) were identified. Two FBs were related to broken surgical instruments during orthognathic surgery (a suture needle and a burr fragment), while two were fractured local anesthesia needles during third molar extraction under local anesthesia. All FBs were located in deep compartments of the masticatory space (paramandibular or pterygopalatine region). Surgical retrieval via transoral approach under general anesthesia was successful in all cases. One patient experienced transient facial nerve dyskinesia; no long-term complications or recurrences were noted. **Conclusions**: Retained foreign bodies in the masticatory space are infrequent yet warrant prompt recognition and surgical management to mitigate the risk of infection, nerve damage, and repeated procedures. Thorough instrument checks, proper technique, and advanced imaging modalities are crucial for minimizing these complications in orthognathic surgery.

## 1. Introduction

The masticatory space is a complex anatomical and functional entity primarily centered around the mandibular ramus, which divides it into medial and lateral compartments [1]. This space is bordered by other anatomic regions, including the oral cavity anteriorly, the parotid space posteriorly, the parapharyngeal space medially, the paramandibular and sublingual spaces inferiorly, and the skull base superiorly. The masticatory space houses the muscles of mastication (masseter, medial pterygoids, lateral pterygoids, and temporalis), the mandibular and maxillary branches of the trigeminal nerve, and several vascular structures. The deep location and intricate anatomy of the masticatory space present unique challenges in clinical examination and treatment [2]. Delayed detection and complex management of lesions in this region are not uncommon and may carry significant prognostic implications in head and neck cancers [3]. Foreign bodies (FBs) in the masticatory space are rare but clinically significant events, often resulting from dental and oral surgical procedures [4,5]. These include broken needles, suture materials, and other surgical instruments that can penetrate or become embedded in the soft tissues [6]. Such incidents are usually accompanied by symptoms like pain, swelling, trismus, and purulent discharge [7]. The mobility of the masticatory space, coupled with the proximity of vital anatomical structures, makes the management of these FBs critical to prevent serious complications [8,9].

The precise yet invasive maneuvers required in orthognathic surgery can elevate the risk of instrument failure, leading to the inadvertent retention of FBs. To the best of our knowledge, two cases of retention of FBs—surgical bur—after orthognathic surgery have been reported in the literature up to now [10].

The present manuscript aims to summarize our experience in the diagnosis and treatment of FBs within the masticatory space, focusing on the cases retrieved in orthognathic surgery procedures. Diagnostic and surgical strategies, key lessons, and follow-up protocols emerging from the presented case series are outlined. Our focus was on highlighting the challenges encountered to provide insights into effective management strategies for treating similar cases in the future.

## 2. Case Series

This study was conducted in compliance with ethical standards and guidelines. Ethical approval for the research was obtained from the Clinical Institutional Review Board at the University of Siena (Reference Number: 7/2023; approval date: 7 October 2023). The study adhered to the principles outlined in the Declaration of Helsinki regarding research involving human subjects. Written informed consent was obtained from the patients for the use of their clinical data.

We performed a comprehensive and systematic search of the medical database of the Department of Medical Biotechnologies, Maxillofacial Surgery Unit, University of Siena. This database includes structured entries for all surgical procedures, operative reports, radiologic imaging, diagnostic codes, and follow-up documentation. The search period extended from 1 January 2017, up to 31 December 2024. The primary search keywords employed were “foreign body” and “masticatory space.” Patients were included if they met the following criteria: (i) referring for orthognathic surgery; (ii) undergoing dental procedures associated with orthognathic surgery, such as third molar extraction in patients with malocclusion scheduled for corrective jaw surgery; (iii) complete clinical and radiological data. The data extraction process focused on the methodologies employed for the diagnosis and surgical removal of FBs, the types of foreign bodies encountered, complications reported, and the follow-up.

Out of 2092 surgical procedures performed in the searched period, we identified four patients who met these criteria (0.19%), all of whom underwent successful surgical retrieval of the FB. Of these, two patients were referred by a dental practitioner to our unit while two inpatients came from our orthognathic surgery activity. Individual characteristics of the retrieved cases are reported below and summarized in Table 1.

All surgical procedures were performed by our surgical unit and the same first operator (PG).

**Figure 1:**
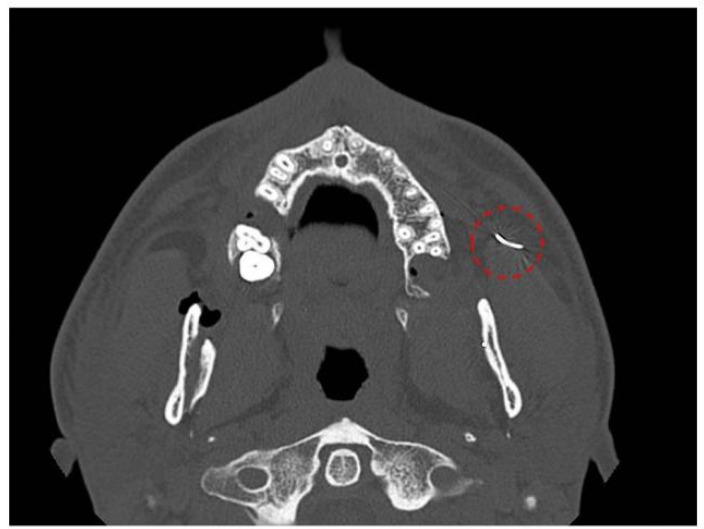
Axial computed tomography (CT) image demonstrating the presence of a suture needle within the left masticatory space. The foreign body is indicated by a dotted circle.

Patient 1: A 21-year-old male patient underwent bimaxillary orthognathic surgery for Class III malocclusion. At the end of the procedure, the loss of a suture needle occurred while closing the upper vestibular incision. Intraoperative attempts to locate and remove the FB failed. Postoperative CT showed the FB located in the left paramandibular space, in relation with the anterior border of the masseter muscle and the parothid gland (Figure 1).

A second surgical procedure was performed two days later to locate and remove the needle. A transoral approach was used, accessing the site through the pre-existing Le Fort I incision. As blunt dissection did not allow direct visualization of the FB, multiple C-arm X-rays were taken while tracking its position with a metallic forceps. This necessitated extending the incision and performing a deeper dissection until the FB was identified and successfully removed. During follow-up, the patient exhibited mild dyskinesia of the facial nerve, limited to perioral motion, with no complete deficit. Symptoms resolved spontaneously within four months. Follow-up included neuromuscular assessments at 1 week, 1 month, 3 months, and 6 months, confirming full functional recovery without long-term sequelae.

**Figure 2:**
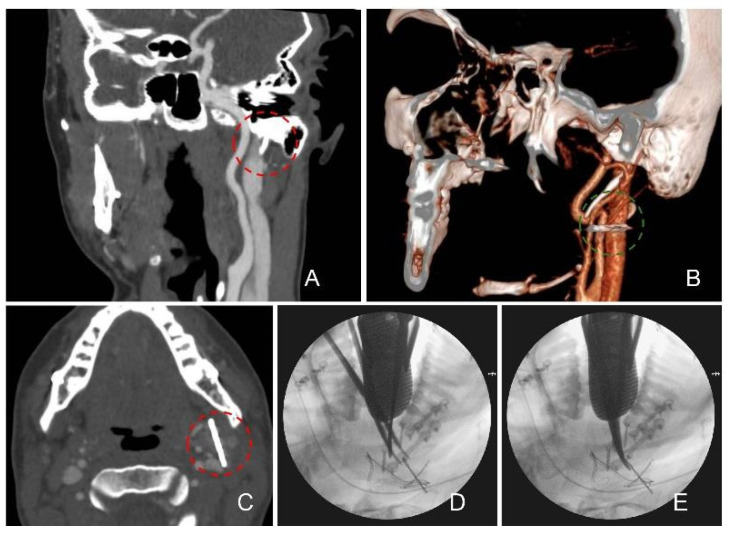
Coronal, 3D and axial Angio CT preoperative images localizing the foreign body in proximity of the right jugular vein (**A**–**C**); foreign body removal with intraoperative C-arm X-ray (**D**,**E**). The foreign body is indicated by a dotted circle.

Patient 2: a 30-year-old male underwent orthognathic surgery for skeletal Class II malocclusion. At the six-month postoperative follow-up, a CT scan was performed in response to the patient’s report of a specific discomfort in the left mandibular region. Imaging unexpectedly revealed a retained FB in the left paramandibular space, adjacent to the parotid gland and jugular vein. The FB was suspected to be either a screw or a bur lost during mandibular osteosynthesis performed as part of the Bilateral Sagittal Split Osteotomy procedure. To plan the surgical removal, a neuronavigation CT scan was performed using the StealthStation system (Medtronic, Dublin, Ireland). Surgery was carried out via a transoral approach through the pre-existing left sagittal split osteotomy incision. As blunt dissection did not allow direct visualization of the FB; thus, a navigation pointer was used to guide localization. Intraoperative C-arm fluoroscopy was employed to confirm the position while tracking the object with a metallic forceps. The FB, ultimately identified and removed, was a fragment of a surgical bur. No major vascular structures were encountered during the procedure (Figure 2).

**Figure 3:**
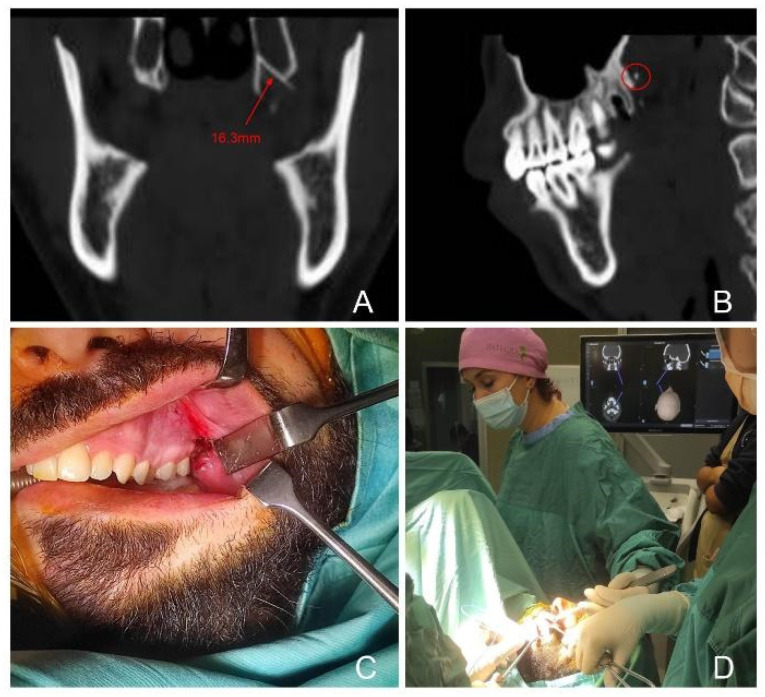
CT Coronal and sagittal scans locating the foreign body in the left pterygopalatine space (**A**,**B**); intraoperative removal from transoral approach and using CT-based intraoperative navigation (**C**,**D**). The foreign body is indicated by a dotted circle.

**Figure 4:**
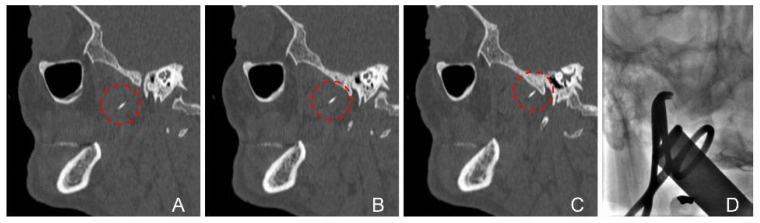
CT sagittal images showing syringe needle in the masticatory space (**A**–**C**) and surgical removal with intraoperative C-arm X-ray (**D**). The foreign body is indicated by a dotted circle.

Patient 3 and 4: Both subjects—32- and 33-year-old male patients—underwent an upper third molar extraction procedure preliminar to orthognathic surgery under local anesthesia. Following the administration of the anesthesia, the dentist identified the broken syringe needle (Figure 3 and Figure 4) and referred the patient to our unit.

CT scan at our hospital revealed a FB in the right pterygopalatine space adjacent to the medial pterygoid muscle in both cases. Both patients were treated under general anesthesia with fluoroscopy X-ray. Moreover, patient 4—that complained of trismus at admission—also required a fibroscopy-guided intubation and intraoperative CT-based electromagnetic navigation (StealthStation, Medtronic, Dublin, Irland), as shown in Figure 4.

In these cases, intraoperative identification of the foreign body was facilitated by the use of a neuronavigation pointer (case 3) and C-arm X-ray imaging (cases 3 and 4). In case 3, the lingual nerve was visualized and successfully preserved. An additional challenge in both procedures was the displacement of surrounding soft tissues caused by forced mouth opening during anesthesia and surgery.

## 3. Discussion and Literature Review

Orthognathic surgery, derived from the Greek words “orthos” (straight) and “gnathos” (jaw), has evolved significantly since its inception in the early 20th century. Initially developed to address severe dentofacial deformities, the field has advanced with innovations in anesthetic and surgical techniques, imaging modalities, computer-assisted planning, and orthodontic integration that have enabled more complex interventions, including bimaxillary surgeries and simultaneous soft tissue corrections [11,12]. Today, orthognathic surgery not only restores functional occlusion but also improves facial aesthetics and quality of life, with applications in both congenital and acquired jaw deformities. Despite these advancements, complications remain inherent to the procedure and require timely and meticulous medical diagnosis and treatment including, in some cases, surgical revision [13]. A systematic review by Jędrzejewski et al. (2015) revealed the following rate of complications among included Randomized Clinical Trials and Clinical Trials: nerve injury/sensitivity alteration (50.00%), temporomandibular joint disorders (13.64%), hemorrhage (9.09%), auditory tube dysfunction and hearing complaints (6.82%), infection (6.82%), bad split (4.55%), nonunion of osteotomy gap (4.55%), skeletal relapse (4.55%), septum deviation (2.28%), bone necrosis (2.28%), soft tissue injuries (2.28%), positional vertigo (2.28%), dental complications (2.28%), postoperative swelling (2.28%), and psychological depression (2.28%) [14]. Among the rare procedural complications that require secondary surgical intervention, FB retention has been specifically addressed in a detailed investigation by [10]. In a retrospective analysis of 76 consecutive orthognathic surgeries, these authors documented surgical bur breakage in five cases (approximately 7% incidence). Of those five occurrences, three fragments were successfully retrieved intraoperatively, while the remaining two were left in situ. Notably, one patient developed a persistent FB reaction about one year postoperatively, ultimately requiring surgical removal of the embedded bur fragment. These findings underscore both the relative frequency of bur breakage—dense cortical bone and repeated instrument sterilization pointed out as possible factors—and the potential for delayed complications [10] highlighting the importance of rigorous intraoperative checks (including instrument inspection and radiographic confirmation) and the need for proactive follow-up. The authors concluded by stating that, when fragments cannot be retrieved, close monitoring becomes critical to identify any emerging FB reaction, infection, or other sequelae that would necessitate a secondary surgical intervention [10].

A useful framework for categorizing and comparing surgical complications is the Clavien–Dindo (CD) classification, which stratifies adverse events based on the degree of intervention required [15]. Specifically, complications requiring a return to the operating room under general anesthesia are classified as Grade IIIb. In the present case series, the retained FBs in the masticatory space necessitated a second surgical exploration under general anesthesia for their retrieval, thereby fitting the criteria for a Grade IIIb complication. Although the overall incidence of such complications in orthognathic surgery is low, recognizing them as CDIIIb underscores their clinical significance: they involve additional morbidity, prolonged hospitalization and recovery time, ultimately leading to added financial costs. To the best of our knowledge, this is the largest report in the literature comprehensively detailing the secondary surgical management of four patients who were diagnosed with retained FBs in the masticatory space following orthognathic surgery or related preliminary procedures. Two of these cases involved instruments lost during the primary surgical procedure—a suture needle and a bur fragment—while the other two involved the breakage of syringe needles during local anesthesia prior to orthognathic surgery. All four instances required a secondary intervention under general anesthesia—lasting 130 to 250 min—to locate and remove the retained objects, classifying them as CDIIIb complications. Notably, imaging—especially CT scans and intraoperative C-arm radiography—played a pivotal role in accurately identifying the FB position and facilitating precise removal. Postoperative outcomes and long-term follow-up were favorable in all cases; none of the patients experienced infection, one patient had transitory facial nerve damage manifested as dyskinesia. These findings emphasize that, while FB retention in the masticatory space remains rare, clinicians should maintain a high index of suspicion, employ meticulous intraoperative instrument checks, and utilize advanced imaging guidance for safe and efficient retrieval when such events occur.

We performed a Scoping review according to PRISMA-ScR criteria. Major medical databases, including PubMed, Scopus, Web of Science, and the Cochrane Database of Systematic Reviews, were reviewed. The search strategy incorporated various keyword combinations—such as “dental needles,” “needle fracture,” “broken dental needle,” and related terms—to ensure broad coverage of relevant studies. Retrieved records were subsequently screened for relevance to the topic of foreign bodies in the masticatory space. Our literature review (summarized in Table 2) underscores the rarity yet clinical significance of retained foreign bodies within the masticatory space since only case reports and small case series were retrieved in the last five years of published literature

Most manuscripts focused on broken anesthetic needles—most commonly 27G or 30G—that either migrated or remained embedded within deep anatomical planes [16,17,18,19,20]. Preoperativelly imaging modalities included CT and panoramic radiography [16,17,18,19,20], and, increasingly, image-guided surgical navigation were routinely employed for intraoperative localization [16,17,18,25]. Surgical retrieval preferred transoral approach [16,18,20,21,22,23] including a case of intraoral endoscope-assisted method [19,20] and two reported cases of transcutaneous procedures [17,24]. Several authors emphasized the importance of avoiding smaller-diameter needles (e.g., 30G) or repeatedly bending needles, as these practices increase the risk of fracture [16,17,19,20,21,23,24,25]. Although complications such as transient nerve deficits (e.g., lingual nerve anesthesia), infection, and trismus were occasionally reported, early detection and prompt surgical intervention generally led to favorable outcomes [16]. Collectively, the literature highlights the importance of meticulous injection technique, vigilant intraoperative instrumentation protocols, and the availability of advanced imaging resources to facilitate successful retrieval of retained FBs. It is crucial to recognize that many routine dental procedures may lead to foreign FBs in the masticatory space. Dental extractions, one of the most commonly performed surgical procedures worldwide, are particularly prone to such incidents especially when an inferior alveolar nerve block anesthesia for impacted third molars is required [5]. With the prevalence of impacted third molars reported to range from 16.7% to 68.6% across various populations [26], the risk of needle breakage in these procedures is significant. Moreover, a survey by Jayadevan et al. (2014) indicated that 65% of U.S. dentists had encountered at least one instance of a lost surgical needle [27]. These findings suggest that the true incidence of FB retention in the masticatory space may be higher than currently recognized, underscoring the need for preventive measures, improved diagnostic vigilance, and proactive intervention.

**Figure 5:**
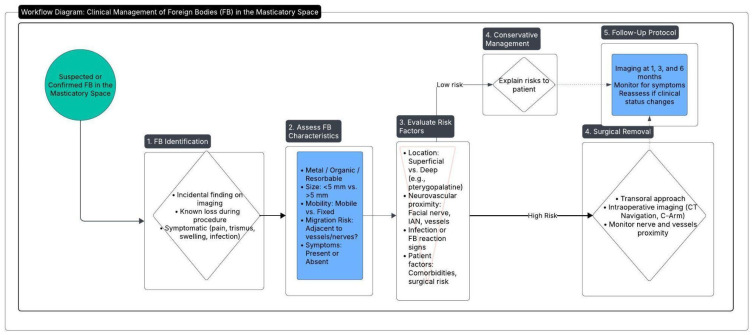
This diagram outlines the process for managing a suspected or confirmed foreign body (FB) in the masticatory space. The 5 steps process includes identifying the FB, assessing its characteristics and risks, and deciding on a treatment approach based on risk assessment.

In our experience, CT navigation—though valuable for preoperative planning and initial localization—did not offer the same flexibility for real-time tracking of foreign bodies that may shift under intraoperative manipulation. In contrast, intraoperative X-ray (also referred to as C-arm X-ray and fluoroscopy) provided immediate visual feedback on any positional changes, thereby facilitating more precise and efficient removal. Nonetheless, repeated use of fluoroscopy raises concerns about cumulative radiation exposure, both for patients and surgical staff. In this context, intraoperative echography (ultrasound) represents a promising but as yet underreported technique for localizing retained foreign bodies during maxillofacial procedures. Ultrasound imaging offers real-time visualization without ionizing radiation, potentially enhancing intraoperative safety and cost-effectiveness [28,29]. Its applicability, however, would likely depend on operator expertise, adequate acoustic windows, and the echogenic properties of the retained fragment [30]. As surgical teams gain proficiency with emerging imaging modalities, the combination of high surgeon experience and advanced, real-time imaging may further reduce complications associated with FB retention in the masticatory space. Moreover, as with any surgical intervention, the experience and expertise of the operating surgeon is paramount. In high-stakes environments such as orthognathic surgery—where deep anatomical structures and critical neurovascular bundles are at risk—surgeons with extensive familiarity in the field are better equipped to prevent and manage rare complications like FB retention. Our proposed flow-chart for diagnosis and treatment of FB in the masticatory space is outlined in Figure 5.

This study is subject to several limitations. First, the retrospective nature and the small sample size—limited to four cases over an eight-year period—reflect the rarity of this complication but also limit the generalizability of the findings. Second, while a comprehensive review of the literature was conducted using PRISMA-ScR principles, the search was not registered in advance in a systematic review database, and formal risk of bias assessments were not performed. Third, although all foreign bodies were surgically retrieved, the study does not include cases in which retained objects may have been left in situ and monitored, potentially underestimating the broader clinical spectrum. Lastly, the single-center design may introduce institutional or operator-related bias, particularly since all surgeries were performed within a highly specialized maxillofacial unit with advanced imaging and navigation resources that may not be available in all settings.

## 4. Conclusions

FB retention in the masticatory space remains an uncommon but clinically significant complication in orthognathic surgery and related procedures. Our case series, as well as the supporting literature, highlights the critical importance of meticulous intraoperative checks, proper instrumentation, and the use of advanced imaging for both diagnosis and retrieval. Although second-stage surgical intervention—often under general anesthesia—is frequently necessary, timely identification and removal of the FB can prevent severe complications such as nerve damage, chronic infection, or granuloma formation. Future efforts should focus on standardized preventive measures (e.g., instrument integrity checks) and continued refinement of image-guided surgical techniques to ensure enhanced patient safety and improved long-term outcomes.

## Figures and Tables

**Table 1:** Clinical, surgical, and follow-up data of patients with foreign bodies in masticatory space.

Patient ID	Age/Sex	Type of FB	Location and Relationship	Symptoms	TTS (Days)	Intubation	Surgical Approach	ST (Minutes)	Adjuvant Equipment	Intraoperative Findings	Outcome	Follow-Up (Months)
Patient 1	21, M	Suture needle	Left paramandibular space, anterior border of masseter muscle	NR	2	INT	Transoral	120	C-Arm X-ray	Facial Nerve	FB Extracted; Facial nerve lesion (dyskinesia)	24
Patient 2	30, M	Bur tip	Left paramandibular space, parotid gland, and jugular vein	None	180	INT	Transoral	180	CT Navigation, C-arm Xray	NR	FB Extracted	6
Patient 3	32, M	Syringe needle	Right pterygopalatine space, medial pterygoid muscle	None	0	INT	Transoral	130	C-Arm X-ray	Lingual Nerve	FB Extracted	NR
Patient 4	33, M	Syringe needle	Right pterygopalatine space, medial pterygoid muscle	Trismus	10	IOT (fibroscopy guided)	Transoral	250	CT Navigation; C-Arm X-ray	NR	FB Extracted	12

Abbreviations: C-Arm X-ray—Fluoroscopy-guided X-ray; FB—Foreign Body; INT—Intubation; IOT—Intraoperative Tracheal Intubation; NR—Not Reported; ST—Surgical Time; TTS—Time to Surgery.

**Table 2:** Summary of literature review on foreign bodies in the masticatory space.

Author, Year [Ref.]	Study Type	Sample Size	Type of Foreign Bodies	Diagnostic Methods	Surgical Techniques	Complications	Key Findings
Lehmann, 2020 [16]	Case series, review	6	4 hypodermic needles (27G, 25G, 30G, 30G) 1 cottonoid surgical sponge 1 maxillary molar	CT, optical navigation system	Transoral, endoscopically assisted and image-guided	Transient lingual and IAN sensory deficits	Minimally invasive approach through endoscopy
Karakida, 2020 [17]	Case report	1	33G, 12 mm needle	Panoramic X-ray, CT, C-arm fluoroscopy	Transcutaneous (neck skin)	None	Avoid using thinner-diameter needles and completely insert the needle
Schorn, 2021 [18]	Case report	1	30G needle	OPTG, CBCT, CT, 3D navigation	Retromandibular	None	Fractured needles can move quickly, early recovery is necessary, and 3D navigational planning immediately
Terada, 2022 [19]	Case Report	1	33G needle	CT, Panoramic X-ray, LL X-ray	Intraoral using Fluoroscopy assisted by endoscopy	None	No smaller 27G, no bending, immediate surgical intervention
Malkawi, 2023 [20]	Case report	1	30G	CBCT, CT with contrast	Intraoral, endoscopically with C-arm X-ray	None	Appropriate local anesthetic technique to avoid needle fracture. The endoscope, image guidance, and the transoral approach can be carefully employed to promote the success of the retrieval technique.
Erdil, 2022 [21]	Case report	1	30G	CBCT, Orthopantomogram	Intraoral	Trismus	Avoid bending, 25–27G, avoid direction changes, inform patient not moving, urgent intervention
Chybicki, 2020 [22]	Case report	1	30G	Periapical radiograph	Intraoral	None	Treating cases involving patients with behavioral disorders, such as autism spectrum disorder, under GA
Dilworth, 2022 [23]	Case report	2	30G, 27G	Panoramic, CT, Cephalometric and panoramic radiograph	Intraoral, intraoperative AP X-ray	None	30G too small, avoid bend and multiple injections and change direction, patient cooperation
Teixeira, 2021 [24]	Case report	1	30G	X-ray, CT	Transcutaneous (neck skin)	None	Avoid needle bending, multiple passes, and patient movement, prefer longer needle 27G for IANB
Sandre, 2021 [25]	Case series	12	NR	C-arm fluoroscopy	NR	NR	Avoid bending needle, rapid removal, establish protocol

Abbreviations: GA—General Anesthesia; AP—Anteroposterior; CBCT—Cone Beam Computed Tomography; CT—Computed Tomography; IAN—Inferior Alveolar Nerve; IANB—Inferior Alveolar Nerve Block; LL X-ray—Lateral-Lateral X-ray; NR—Not Reported; OPTG—Orthopantomogram (Panoramic Radiograph); 3D—Three-Dimensional.

## Data Availability

The raw data supporting the conclusions of this article will be made available by the authors on request.
